# Splenic cord capillary hemangioma with non-islet cell tumor hypoglycemia: a case report

**DOI:** 10.1186/s13000-019-0915-0

**Published:** 2019-12-16

**Authors:** Tatsuaki Daimon, Takeo Kosaka, Minoru Horinaga, Junichi Saito, Yoshito Ueyama, Shoji Matsuzaki, Mototsugu Oya

**Affiliations:** 1 Department of Urology, Keio University School of Medicine, 35 Shinanomachi, Shinjuku-ku, Tokyo, 160-8582 Japan; 2 Department of Urology, Inagi Municipal Hospital, 1171 Omaru, Inagi-city, Tokyo, 206-0801 Japan; 3 Department of Surgery, Inagi Municipal Hospital, 1171 Omaru, Inagi-city, Tokyo, 206-0801 Japan; 4 Department of Pathology, Inagi Municipal Hospital, 1171 Omaru, Inagi-city, Tokyo, 206-0801 Japan

**Keywords:** Insulin-like growth factor-II, Non-islet cell tumor hypoglycemia, Splenic cord capillary hemangioma, Splenic hamartoma, Splenic tumor

## Abstract

**Background:**

Splenic cord capillary hemangioma is a rare benign vascular lesion classified as a splenic hamartoma. On the other hand, non-islet cell tumor hypoglycemia (NICTH) is one of the rare causes of spontaneous hypoglycemia and is considered to be one of the paraneoplastic syndromes. To the best of our knowledge, this is the first reported case of a splenic cord capillary hemangioma with NICTH.

**Case presentation:**

A 25-year-old male was referred to our hospital with hypoglycemia. Except for his low blood sugar, there were no abnormal findings from laboratory tests, which included an endocrinological examination. Enhanced computed tomography confirmed the presence of a solid mass measuring about 6 cm in the retroperitoneum, and a tumorectomy was performed. During this operation, it became clear that the tumor turned out to be a splenic parenchyma, and as a result, a total splenectomy was performed. Microscopically, we diagnosed this as a cord capillary hemangioma, and through immunohistochemistry, we found that some tumor cells were positive for insulin-like growth factor -II. Fortunately, the hypoglycemia-related symptoms disappeared after surgical resection was performed. The patient is still alive and well without evidence of local tumor recurrence 15 years after the operation.

**Conclusions:**

Splenic cord capillary hemangioma, one of the types of splenic hamartomas, is a very rare benign vascular lesion and might be associated with hypoglycemia thought to be NICTH.

## Background

Splenic cord capillary hemangioma is a rare benign vascular lesion. Once classified under splenic hamartoma, cord capillary hemangioma is now differentiated from hamartoma, as its clonality has recently been proven [1]. And it is also a rare lesion, with an incidence of 0.024–0.13% in tumors of the spleen [2]. On the other hand, non-islet cell tumor hypoglycemia (NICTH) is one of the causes of spontaneous hypoglycemia and is considered to be one of the paraneoplastic syndromes. A key mechanism of NICTH is thought to be that tumor cells excessively produce a high molecular weight form of insulin-like growth factor (IGF)-II [3]. Here we report a rare case of splenic cord capillary hemangioma of a 25-year-old male considered as NICTH. To the best of our knowledge, this is the first case of splenic cord capillary hemangioma with NICTH.

## Case presentation

### Clinical history

This patient was a 25-year-old male who presented with pale skin and sweating to our hospital. His blood glucose level was found to be in the range of hypoglycemia (52 mg/dL), and his symptoms resolved after intravenous dextrose administration. He had experienced this type of episode twice before he visited our hospital but did not have any problems during the previous few days. He had no family history of any endocrine metabolic diseases, and physical examinations showed no special findings. The patient was admitted on the basis of hypoglycemia for 2 weeks. Except for the blood sugar episode, laboratory results regarding complete blood count, routine blood chemistry, kidney function tests, urine analysis, and chest radiograph were all within normal limits. Fasting and oral glucose tolerance load tests were performed, and there were no evidences of the irregular secretion of insulin. Adding to the puzzle, blood glucose levels were within normal limits in the hospital.

**Fig. 1:**
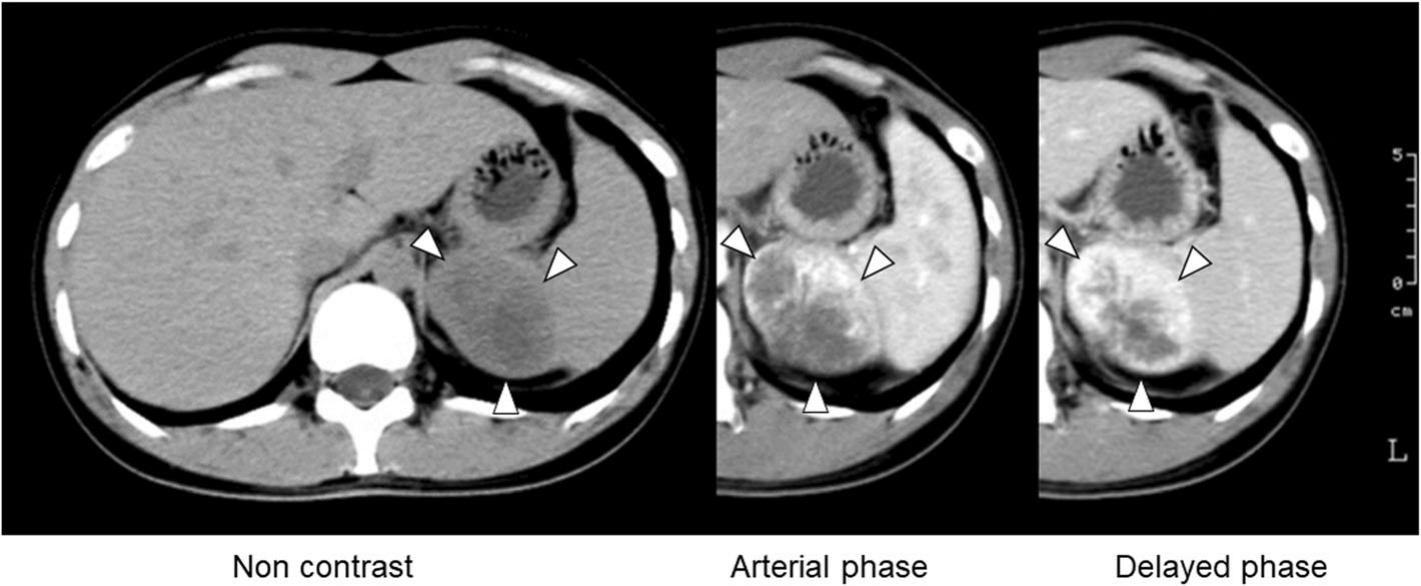
Contrast-enhanced computerized tomography (CT) image. Enhanced computed tomography confirmed the presence of a solid mass measuring about 6 cm with well-defined and regular borders in the retroperitoneum. Peak contrast enhancement in a part of the tumor was seen in the delayed phase

However, enhanced computed tomography (CT) confirmed the presence of a solid mass measuring about 6 cm with well-defined and regular borders in the retroperitoneum. Peak contrast enhancement in a part of the tumor was seen in the delayed phase. The mass seemed to arise from the adrenal gland or kidney, and there were no metastatic lesions (Fig. 1). An MRI study of the mass showed a low signal in T1WI and a high signal in T2WI. As a result of the CT, we made additional endocrinological studies, and the results revealed normal outcomes for serum aldosterone, metanephrine, and normetanephrine and also for 24-h urinary catecholamines and metanephrines. For the tumor located in the retroperitoneum, a tumorectomy was performed. In the course of this operation, there was no evidence of a tumor in the kidney or adrenal gland, but tumor turned out to be in the splenic parenchyma as found by intraoperative ultrasonography. As a result, a total splenectomy was performed.

Hypoglycemia-related symptoms disappeared after surgical resection was performed. The patient is still alive and well without evidence of local tumor recurrence 15 years after the operation.

### Pathological findings

**Fig. 2:**
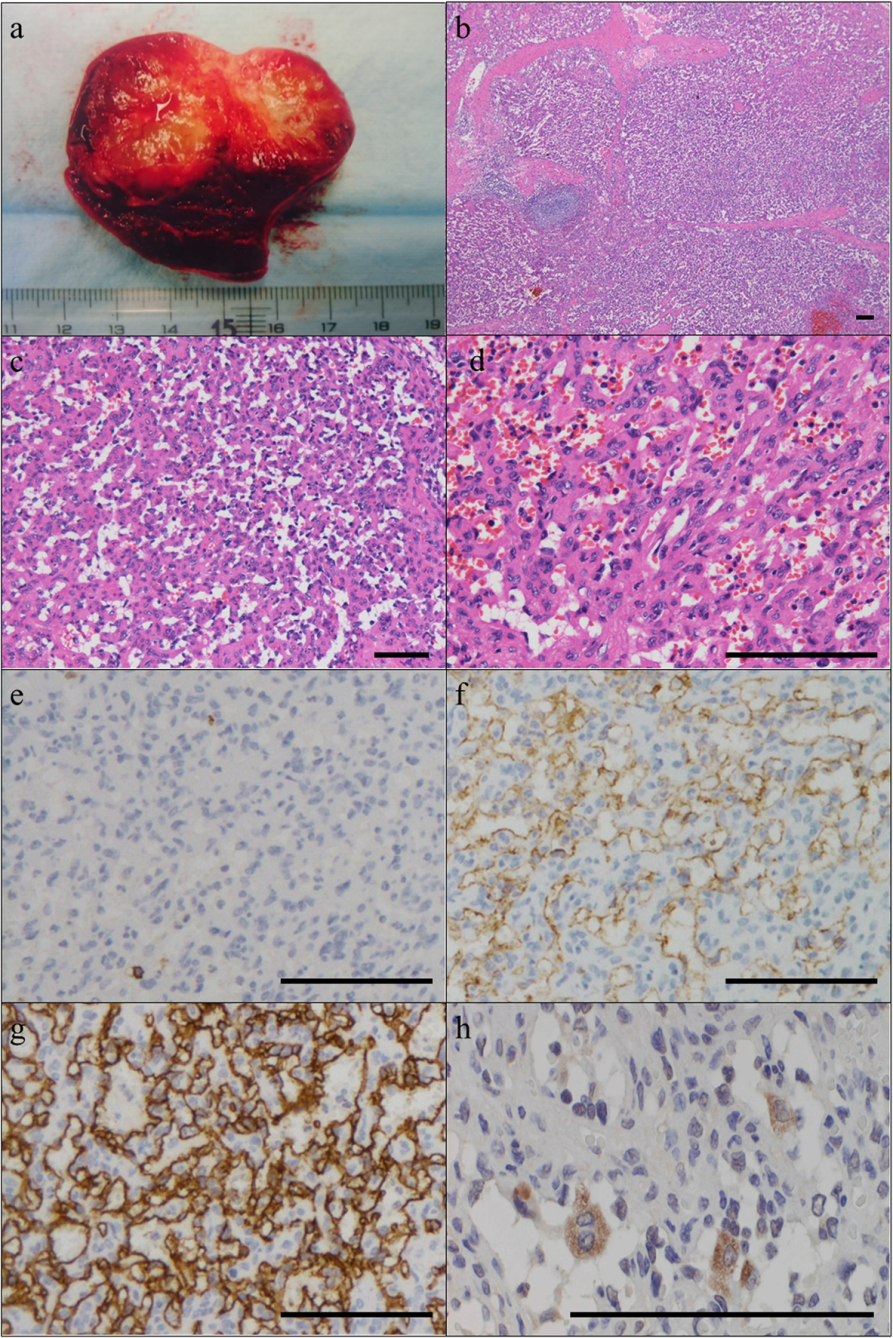
Macroscopic and microscopic findings. (**a**) Macroscopic findings. The solid tumor (3.5 × 2.7 × 2.5 cm) was encapsulated by spleen parenchyma, and the cut surface was a yellowish white. (**b, c**) Microscopic findings on hematoxylin and eosin (HE) staining. Each lobular lesion was surrounded by fibrous tissue and filled with blood cells (B HE × 40, C HE× 100). (**d**) The lesions consist of many vascular channels (HE × 100). Immunohistochemical findings of the lesion. (**e**) Negativity of capillaries and small veins for CD8 (× 100) and (**f**) positivity for CD31 (× 100). Only lymphocytes were positive for CD8 (**e**). (**g**) Positivity of capillaries for CD34 (× 100). (**h**) Some tumor cells were positive for IGF-II (× 400). Bar scale, 100 μm. (b-h)

**Fig. 3:**
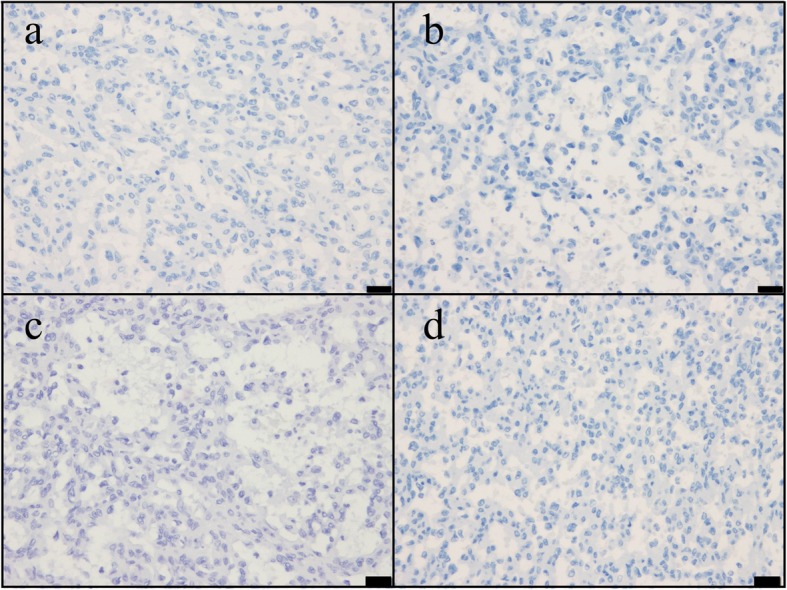
Immunohistochemical findings: synaptophysin(**a**), chromogranin A(**b**), insulin(**c**) and Ki67(**d**). Immunohistochemically, the tumor is negative for synaptophysin, chromogranin A and insulin and less than 1% positive for Ki67. Bar scale, 20 μm. (all)

Grossly, the resected spleen (130 g: 10 × 6.5 × 4 cm) was received. The solid tumor (3.5 × 2.7 × 2.5 cm) was encapsulated by spleen parenchyma, and the cut surface was a yellowish white (Fig. 2a). Microscopically, the tumor showed expansive growth and could be easily distinguished from splenic parenchyma; while fibrous capsule was absent, necrosis was present at the center of the tumor. A histological examination showed that the lesions consisted of many vascular channels that had a striking lobular appearance and were surrounded by fibrous tissue (Fig. 2.b-d). Immunostaining shows a predominance of CD8− (Fig. 2e), CD31+ (Fig. 2f), CD34+ cord capillary (Fig. 2g), and with few sinuses. On the basis of these findings, we finally diagnosed this situation as a cord capillary hemangioma. Moreover, using immunohistochemistry, we found that some tumor cells were positive for IGF-II (Fig. 2h). Moreover, the tumor was negative for insulin synaptophysin, chromogranin A and less than 1% positive for Ki67 (Fig.3).

## Discussion

Splenic cord capillary hemangioma is classified as splenic hamartoma, which is a rare benign vascular lesion with an incidence of three cases in 200,000 splenectomies [4]. Krishnan proposed a classification of splenic hamartomas based on the relative proportion of the various red pulp elements [2] – classic hamartoma and three variant forms: cord capillary hemangioma, myoid angioendothelioma, and the histocyte-rich variant. Immunohistochemically, splenic cord capillary hemangioma is characterized by predominance of CD8 negative, CD31 positive, CD34 positive cord capillary and with few sinuses. We searched cases thought to be splenic cord capillary hemangiomas by PubMed using key words “spleen,” “cord capillary hemangioma,” or “splenic cord capillary hemangioma.” Only a few citations in English were evident. There appear to be few reports about splenic cord capillary hemangioma, although in recent years, over 100 of cases of splenic hamartomas have been published. This situation might have arisen because immunohistochemical studies are needed to precisely classify the types of splenic hamartoma. In 2015, Tajima et al. [1] mentioned that, when strictly defined, there were eight cases of splenic cord capillary hemangioma that were reported before them. After the report, there was only one report about splenic cord capillary hemangioma. Of all the 10 cases documented to date including our case, we were able to obtain 4 cases of clinical courses in detail because 7 cases reported by Chiu et al. [5] had no clinical courses. We have summarized the previous four case reports about splenic cord capillary hemangioma in Table 1.

**Table 1:** Clinical features of four cases of splenic cord capillary hemangioma

case	gender/age	symptoms	PH	Tumor		treatment	follow-up	recurrence	reference
				single or multiple	size				
1	F/35	fever, arthralgia^a^	no	single	30 mm	splenectomy	N/A	N/A	[6]
2	M/45	none	GIST	single	60 mm	splenectomy	8 years	no	[1]
3	M/68	none	HCC	multiple	3-14 mm	splenectomy	N/A	N/A	[7]
4	M/25	hypoglycemia	no	single	60 mm	splenectomy	15 years	no	our case

^a^It was not clear whether those symptoms were caused by the splenic tumor in the report

Generally, it is difficult to find small splenic tumors because there are usually no specific symptoms when the tumor is tiny. However, with its growth or progression, the patient may notice an abdominal mass and experience abdominal distension, back pain, dyspnea, and constipation. Some patients have experienced nontraumatic rupture of the spleen and hypersplenism, such as thrombocytopenia and anemia, even though the splenic cord capillary hemangioma showed no specific symptoms [2]. In our present case, the tumor was detected in its very early stage because the patient received a thorough examination in our effort to resolve his hypoglycemia-related symptoms and indeed not due to symptoms of the tumor’s progression or growth.

Hypoglycemia can be induced by tumors, including pancreatic tumors that secrete insulin, and also by non-islet cell tumors that secrete IGFs. Therefore, in the absence of insulinoma, NICTH should be considered. NICTH is thought to be related to the production and secretion of IGF-II by the tumor [3]. NICTH is one of the causes of spontaneous hypoglycemia. In some cases, patients experience severe hypoglycemia, and intensive treatments such as continuous glucose injections are needed. One of the mechanisms of NICTH is thought to be an excessive production of the high molecular weight form of IGF-II by tumors [3, 8]. In cases of NICTH, it is thought that within tumor cells, the enzymatic processing of pro IGF-II into mature IGF-II is somehow not performed normally. Therefore, tumors secrete incompletely processed pro IGF-II as a high molecular weight form of IGF-II [8].

Most commonly, NICTH has been observed in patients with solid tumors that have either a mesenchymal origin, like fibrosarcoma, fibroma, and mesothelioma, or which have an epithelial origin, such as hepatocellular carcinoma and adrenocortical carcinoma. These tumors are frequently quite large at the time of diagnosis. About 70% of them have been greater than 10 cm in diameter [9]. In our present case, the patient fortunately experienced hypoglycemia-related symptoms that were relatively mild and temporary. This might have been because the size of the tumor was smaller than tumors reported before.

One of the limitations of this case report is that we did not detect the production of the high molecular weight form of IGF-II in his serum, and therefore, it was not certain whether this NICTH case was related to the high molecular weight form of IGF-II. In this particular case, this NICTH might have been related to the high molecular weight form of IGF-II since serum growth hormone and plasma cortisol were relatively low (GH 0.15 ng/mL (< 1.46 ng/mL) and the cortisol level was 16.6 μg/dL (4.0–23.3 μg/dL) [8]. Moreover, we confirmed the presence of IGF-II-positive cells in the tumor by an additional immunohistochemistry. To the best of our knowledge, there has not previously been a report in the literature about a case of splenic cord capillary hemangioma with NICTH.

## Conclusions

We reported a case of a splenic cord capillary hemangioma with NICTH. This is especially noteworthy since cases of splenic cord capillary hemangioma are extremely rare and more so in combination with NICTH. Because of the hypoglycemia-related symptoms in this case, the tumor was found in the early stage. For pathologists, careful histological examination is necessary not only to make a correct diagnosis with splenic cord capillary hemangioma but also to evaluate the presence of the high molecular weight form of IGF-II when the patients have hypoglycemia-related symptoms and consider the possibility of NITCH.

## Data Availability

All data generated or analyzed during this study are included in this published article.

## Abbreviations

CCH: Cord capillary hemangioma
CD: Cluster of differentiation
CT: Computed tomography
GIST: Gastrointestinal stromal tumor
HCC: Hepatocellular carcinoma
HE: Hematoxylin and eosin
IGF: Insulin-like growth factor
MRI: Magnetic resonance imaging
N/A: Not available
NICTH: Non-islet-cell tumor hypoglycemia
PH: Past history
